# Unraveling the hydrodynamics of split root water uptake experiments using CT scanned root architectures and three dimensional flow simulations

**DOI:** 10.3389/fpls.2015.00370

**Published:** 2015-05-29

**Authors:** Nicolai Koebernick, Katrin Huber, Elien Kerkhofs, Jan Vanderborght, Mathieu Javaux, Harry Vereecken, Doris Vetterlein

**Affiliations:** ^1^Department of Soil Physics, Helmholtz Centre for Environmental Research (UFZ)Halle, Germany; ^2^Agrosphere (IBG-3), Forschungszentrum Jülich GmbHJülich, Germany; ^3^Department of Earth and Environmental Sciences, KU LeuvenLeuven, Belgium; ^4^Earth and Life Institute/Environmental Sciences, Université Catholique de LouvainLouvain-la-Neuve, Belgium

**Keywords:** split-root, R-SWMS, root water uptake, plant root growth, *Vicia faba*

## Abstract

Split root experiments have the potential to disentangle water transport in roots and soil, enabling the investigation of the water uptake pattern of a root system. Interpretation of the experimental data assumes that water flow between the split soil compartments does not occur. Another approach to investigate root water uptake is by numerical simulations combining soil and root water flow depending on the parameterization and description of the root system. Our aim is to demonstrate the synergisms that emerge from combining split root experiments with simulations. We show how growing root architectures derived from temporally repeated X-ray CT scanning can be implemented in numerical soil-plant models. Faba beans were grown with and without split layers and exposed to a single drought period during which plant and soil water status were measured. Root architectures were reconstructed from CT scans and used in the model R-SWMS (root-soil water movement and solute transport) to simulate water potentials in soil and roots in 3D as well as water uptake by growing roots in different depths. CT scans revealed that root development was considerably lower with split layers compared to without. This coincided with a reduction of transpiration, stomatal conductance and shoot growth. Simulated predawn water potentials were lower in the presence of split layers. Simulations showed that this was related to an increased resistance to vertical water flow in the soil by the split layers. Comparison between measured and simulated soil water potentials proved that the split layers were not perfectly isolating and that redistribution of water from the lower, wetter compartments to the drier upper compartments took place, thus water losses were not equal to the root water uptake from those compartments. Still, the layers increased the resistance to vertical flow which resulted in lower simulated collar water potentials that led to reduced stomatal conductance and growth.

## Introduction

Water scarcity is an important abiotic limitation to plant growth and agricultural productivity. Under water limited conditions, changes in root system architecture (RSA) play a major role to reach locations where water is still present, which is often the subsoil. There is no simple relationship between the amount of roots present in certain locations and the actual root water uptake (RWU) from these sites (Pohlmeier et al., 2008). RWU is repeatedly described as a sink moving down the profile with time, only weakly related to root length density in a certain depth (Hainsworth and Aylmore, 1986; Pierret et al., 2003; Garrigues et al., 2006). In many of these studies change in soil water content in a certain depth is assumed to be synonymous with root water uptake. The illustrative Martini glass analogy first used by Zwieniecki et al. (2002) demonstrates that this assumption is too simple. When drinking a sip of Martini with a straw, the Martini is taken up from the bottom of the glass, but a change in “Martini content” is only observed in the upper layer of the glass due to the very high hydraulic conductivity within the glass. Roots and soil matrix are much more complex than the Martini-glass system; however, in soil-plant system the soil hydraulic conductivity and resulting soil hydraulic redistribution also obstruct the view on the site of root water uptake and its temporal dynamics. This has been known for a long time and a number of strategies have been developed to overcome this problem.

An experimental strategy to prevent soil hydraulic redistribution is to divide the root zone into different compartments, which prevent water flow between compartments to permit controlled heterogeneous distribution of soil moisture (Drew, 1975; Herkelrath et al., 1977). In case of horizontal splits, the split layers should additionally be penetrable by roots, which can be, for example, achieved by applying wax or paraffin. When roots take up water in a given compartment the change in total water content can be directly related to root water uptake from this compartment. This assumption can, however, only be drawn if the split layers are completely hydraulically isolated. In the case of water redistribution through the layers, the leakage rate has to be known. Another problem to determine RWU from a soil compartment arises due to the non-linearity of the soil water retention curve. Water content or soil water potential is usually measured at discrete points in the soil. When roots take up water from the soil, strong gradients in soil water potential can develop around the roots. Thus, an extrapolation between point measurements to the complete soil compartment becomes erroneous. A second experimental strategy is to directly observe water flux in soil as it has been successfully demonstrated by Zarebanadkouki et al. (2012). They imaged water flow into roots using neutron imaging of deuterated water. However, this method is hitherto either constrained to quasi two-dimensions (rhizotrons) or very small root systems and to short time scales.

An alternative approach is to quantify the amount of water being translocated by root or soil hydraulic redistribution. Mechanistic root water uptake models that describe water flow in soil, into, and within roots allow quantifying and locating root water uptake and redistribution of water within the soil and root system. The use of mechanistic models, like R-SWMS (root-soil water movement and solute transport, Javaux et al., 2008), has two prerequisites: (i) that the dominant processes are known and (ii) that the required input parameters are available. To fulfill the latter, dynamic information about RSA as well as hydraulic properties of individual root segments have to be available.

RSA has been obtained in the past using root growth models, i.e., RSA is artificially created based on a set of crop specific parameters and rules (e.g., branching rules, growth rates, etc.) derived from experiments (Clausnitzer and Hopmans, 1994; Lynch et al., 1997; Pagès et al., 2004; Leitner et al., 2010). Mostly, one or several typical realizations of RSA obtained from such models for a plant of a certain age have been used to calculate different scenarios, like root water uptake from saline soils (Schröder et al., 2013), performance of varying root architectural traits under different soil moisture regimes (Leitner et al., 2014), or the impact of stomatal regulation type on root water uptake (Huber et al., 2014).

Root growth models have been used as an alternative to 3D-data of root systems as these were not available in the past. However, such data are now becoming increasingly accessible with non-invasive methods reaching a level of resolution which is sufficient to visualize most or all of the root system. The most advanced techniques for imaging soil-grown roots include X-ray computed tomography (Mooney et al., 2012), neutron radiography (Oswald et al., 2008), magnetic resonance imaging (Pohlmeier et al., 2008), or transparent soils (Downie et al., 2012). These techniques are of particular interest because they allow for repeated measurements. When ionizing radiation is used, it is however important to choose appropriate scan parameters to minimize potential damage to living tissues (Dutilleul et al., 2005; Zappala et al., 2013). Previous studies clearly demonstrated the potential of X-ray CT to analyze the temporal dynamics of growing roots (Jenneson et al., 1999; Gregory et al., 2003; Lontoc-Roy et al., 2005). While these early studies were limited to young seedlings, more recent work shows that the same is possible for considerably older root systems (Han et al., 2008; Tracy et al., 2012; Koebernick et al., 2014). First modeling approaches based on the use of RSA from non-invasive imaging are available (Stingaciu et al., 2013). The second challenge remains, i.e., the scarcity of data on root hydraulic properties. Measured data are primarily from hydroponically grown very young root systems. Certain assumptions have to be made to separate radial and axial conductivity during the measurements. Nevertheless, there is a wealth of information on how conductivity changes during root development and these have been used to scale the conductivity of individual root segments (Doussan et al., 1998, 2006). As roots age the resistance in the axial pathway typically decreases due to the maturation of xylem vessels, while in the radial pathway resistance increases with the development of apoplastic barriers (Frensch and Steudle, 1989; Bramley et al., 2009).

In order to avoid confounding root water uptake and hydraulic redistribution by the interpretation of local changes in soil water content we have chosen two of the above strategies: (i) an experimental approach of introducing barriers to avoid soil hydraulic redistribution; (ii) a modeling approach which takes soil and root hydraulic redistribution into account.

The objective of the current study is to compare experimental (introducing barriers to avoid soil hydraulic redistribution) and modeling approaches (calculation of soil and root water flow) with respect to their capacity to localize root water uptake in the presence of strong gradients in soil water potential. Local changes in soil water content will be compared to measured and modeled root water uptake.

For the experimental approach we combined a classical set up using wax barriers (Drew, 1975) with quantitative measurement of RSA over time via X-ray CT. This setup allowed the observation of the relation between RSA and water uptake and how it is affected by soil drying. The addition of paraffin layers allowed for the development of strong spatial heterogeneities in soil water potential, as is generally the case under field conditions.

For the modeling approach we used the mechanistic 3D model R-SWMS (Javaux et al., 2008), which enables a detailed description of soil and root water flow. While R-SWMS so far has only been applied for static (non-growing) root systems, mostly created by root architectural models, we now extended the existing model by an additional root development module, which uses the measured CT-data of RSA over time. Doussan's concept of changing axial and radial conductivity with age (Doussan et al., 2006) was included by using his root hydraulic parameterization by assigning these parameters to root age classes derived from the time lapse 3D RSA CT-Data.

Apart from modeling the actual experimental setup, root distributions obtained from split experiments were also used in simulations without splits and vice versa. This approach allowed us to (i) reinterpret measurement results, (ii) show the influence of split layers on plant water potentials that could be linked to differences in plant/root growth and eventually on root water uptake and (iii) show where soil water is taken up during root growth.

## Materials and methods

### Experiments

Two subsequent experiments under the same environmental conditions (growth chamber, 23°C day/18°C night, 65% relative humidity, photoperiod of 14 h, photon-flux density of 350 μmol m^−2^ s^−1^) were conducted with *Vicia faba* L. cv. Fuego.

The first experiment (3 replications), which will be referred to as “NoSplit” in the following, was conducted with homogeneously filled soil columns of 21.5 cm height with unrestricted soil water flow. The second (4 replications), referred to as “Split” was similar to the first one, but paraffin layers at 5, 10, and 15 cm height were established to interrupt soil water redistribution. This method was adopted from Drew (1975), who showed that root growth was unaffected by such layers. Both experiments were conducted consecutively, which explains the differences in the two setups.

#### Experimental setup

##### “NoSplit” (without paraffin layers)

The porous substrate was prepared by mixing quartz particles of different size classes, consisting of 85% sand, 10% silt, and 5% clay (Vetterlein et al., 2007). Additionally 50 g kg^−1^ of gravel (2–3 mm Ø) and 20 g kg^−1^ of plastic beads (polypropylene, 2–3 mm Ø) were added to the substrate as internal reference for digital image analysis.

**Figure 1:**
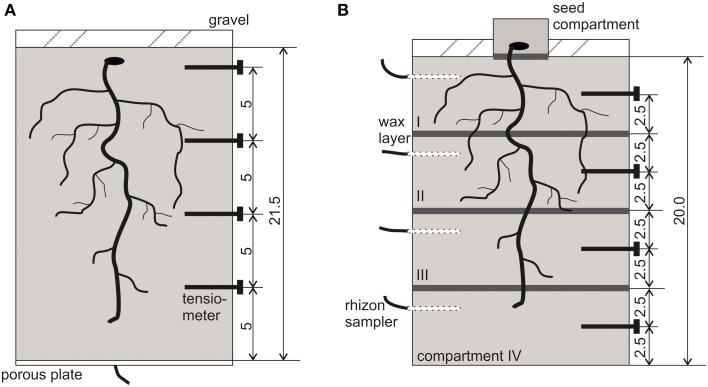
**Schematic view of the experimental setup with locations for tensiometers and paraffin layers. (A)** NoSplit setup, **(B)** Split setup. All dimensions are given in cm.

PVC cylinders (inner Ø = 12.5 cm, h = 21.5 cm) were filled up with the substrate by passing it through two sieves of 4 mm mesh size separated by a distance of 10 cm. This procedure was chosen to avoid particle size separation during filling. Resulting bulk density of the substrate was 1.52 ± 0.01 g cm^−3^. The cylinders had porous plates at the lower end (Figure 1A), which were connected with plastic tubing to a water source. The soil was gently watered with a nutrient solution (modified from Römheld and Marschner, 1990) by capillary rise from the bottom of the sample (soil water potential ψ = 0 hPa at *z* = −21.5 cm). Average volumetric soil water content (θ) at the start of the experiment was 31.1 ± 1%. *Vicia faba* seeds were surface sterilized in 10% H_2_O_2_ solution for 10 min, thoroughly rinsed in deionised water and subsequently imbibed for 1 h in a saturated CaSO_4_solution. Seeds were placed on wet blotting paper and placed in a dark cabinet at room temperature for 2 days. For each cylinder, one pre-germinated seed was carefully placed in a prepared cavity in the soil at a depth of 1 cm. The soil surface was covered by a 2 cm layer of fine quartz gravel. Until shoot emergence columns were covered with aluminum foil to further minimize evaporation. With the removal of aluminum foil the drying period was initiated (Day 6).

##### “Split” (with paraffin layers)

The substrate was the same as in the “NoSplit” experiment, however, without the addition of plastic beads as these caused problems in the segmentation procedure (see below). Soil bulk density was slightly higher (Δ = 0.12 g/cm^3^).

For the split layers, molten paraffin was casted and flattened to a thickness of approximately 0.5 mm and cut into a circular shape. At -5, -10, and -15 cm depth a layer of paraffin was placed on top of the soil and sealed to the cylinder walls using molten paraffin (Figure 1B). For initial irrigation, we placed rhizon soil moisture samplers (Eikelkamp, Giesbeek, NL) in each soil compartment. Those were connected over night to bottles filled with 150 ml nutrient solution each. Volumetric water content at the start of the experiment was 23.8 ± 0.5% in each compartment. Seed preparation was the same as in the “NoSplit” experiment. To avoid the formation of cracks in the soil due to the placement of large *Vicia faba* seeds, these were planted in a separate seed compartment: a cylinder (Ø = 6 cm, h = 3 cm) filled with the soil mixture and 20 ml of water. When the roots emerged through the paraffin layer at the bottom of the seed compartment, the small cylinder was placed on the topsoil (Day 0). The remaining bare topsoil was covered with gravel to reduce evaporation. The split samples were initially also covered with aluminum foil, which was removed on Day 4 to start the drying period.

#### Transpiration and soil matric potential

The PVC cylinders were placed on weighing cells (KERN 572, Kern and Sohn GmbH, Balingen, Germany), and grown for 30-36 days with no additional watering. Weight data were recorded every 10 min throughout the experimental period. Four micro-tensiometers (Vetterlein et al., 1993) were inserted horizontally through sealed boreholes (“NoSplit”: -1.5, -6.5, -11.5, and -16.5 cm soil depth; “Split”: -2.5, -7.5, -12.5, -17.5 cm, Figure 1) to monitor the soil matric potential (ψ_m_), during drying.

The daily transpiration rate was calculated from weight differences between two subsequent days. Evaporation was assumed to be negligible due to the layer of coarse gravel on the surface and as surface was never rewetted during the experiment. Relative humidity was constant day and night hence dew formation could also be excluded. Only on the seed compartment used in “Split” experiment, there was no gravel layer and hence water applied initially (20 ml) was assumed to be lost by evaporation uniformly within the first 7 days.

Leaf area development was estimated by daily measuring the length and width of the lamina of each leaflet and using the linear model of Peksen (2007):

(1) LA = 0.919+0.682 L ∗ W

where *LA* [cm^2^] is the one-sided leaf area, *L* [cm] is the length of the lamina, and *W* [cm] is the width of the lamina. After harvest, we used a flatbed scanner to measure leaf area. The results agreed well with the estimation using Peksen's model. Stomatal conductance was measured at the end of each day using a steady-state porometer (SC-1 Leaf Porometer, Decagon Devices, Inc., Pullman, WA, USA). Two measurements per plant were taken on the abaxial side of the youngest unfolded leaf pair and the mean value of the two measurements was stored.

#### CT scanning and image analysis

All samples from the “NoSplit” and the “Split” experiment were scanned every second day during the night phase with an industrial X-ray micro-CT scanner (X-Tek HMX 225) with a fine focus X-ray tube. The scanning parameters are summarized in Table 1. Potential X-ray dose was estimated using the free online tool Rad Pro Dose Calculator (McGinnis 2002-2009). In the “Split” experiment, which had a higher exposure, cumulative dose at the end of the experiment was 4.8 Gy. This is well below the maximum dose (approximately 30 Gy) suggested for plant CT studies by Zappala et al. (2013). Due to the height of the cylinders separate scans of the upper and the lower part of the sample had to be performed. In the NoSplit setup the mechanism for attaching the porous plate to the soil cylinder at the bottom required an additional plastic ring for sealing reasons which caused photon starvation at the lower end (7 cm), so that not the entire root system could be imaged.

**Table 1:** **Table 1 X-ray settings used in the different experimental setups**.

	**NoSplit**	**Split**
Voltage [kV]	200	210
Current [μA]	250	500
Number of Projections [-]	800	2000
Exposure time [ms]	200	200
Resolution [μm]	245	277

**Figure 2:**
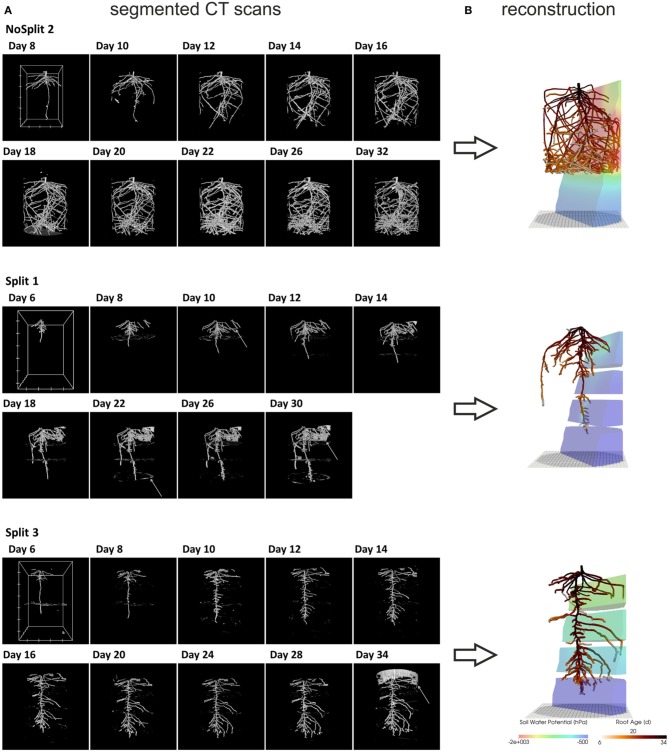
**(A)** Three dimensional rendered view of the segmented CT images at different scan times. White arrows indicate misclassified objects: NoSplit 2, Day 8: plastic bead Split 1, Day 10: tensiometer, Day 22: paraffin layer, Day 30 soil crack. Split 3, Day 34: container wall. White boxes at Day 8 or 6 show the scaling of the root system: the distance between two ticks equals 100 pixels, which equals 2.45 cm for NoSplit2 and 2.77 cm for the Split setups. **(B)** VR reconstructions of root system architectures at the end of each experiment within their respective soil Root systems are colored according to root age and the soil according to the simulated soil water potential.

Although the samples were positioned carefully, images scanned at different times were not perfectly aligned. A manual, feature-based method was used to register the images (see Koebernick et al., 2014). The scans from the upper and lower halves of the samples were combined into a single image. The raw images were filtered with a total variation filter (Rudin et al., 1992) to remove small scale noise while preserving sharp edges. We additionally used a pseudomedian filter (Pratt, 1991) to enhance the contrast between roots and soil and to remove beam hardening artifacts. Roots were segmented from the background using a region growing algorithm, similar to the approach of Kaestner et al. (2006). The algorithm used two thresholds to determine, whether a voxel belongs to the root system. The thresholds were chosen manually based on the histogram and visual inspection of the segmentation results. The images were processed with the freely available software QtQuantim (www.quantim.ufz.de). A more detailed description of the technical procedure can be found in Koebernick et al. (2014). In the NoSplit experiment, two samples (NoSplit 1 and NoSplit 3) could not be successfully segmented due to technical difficulties. Due to improved scanning conditions for the Split setup all architectures could be segmented. The segmented images of the root systems are shown in Figure 2A. These images contained a number of misclassified voxels (e.g., wall material, paraffin layers, cracks, tensiometers) and roots were disconnected at some points.

For the subsequent simulations, a connected root structure was required. Thus, the binary images had to be manually reconstructed using a three-dimensional virtual reality system, which was initially developed to reconstruct MRI data but can be used for any binarized images (for a detailed description of this method see Stingaciu et al., 2013). Due to the labor-intensive manual reconstruction only two replications of the “Split” (Split 1 and Split 3) experiment were reconstructed. We chose Split 1 and Split 3 because these cover the contrasting root architectures in the “Split” experiment. Misclassified regions in the binarized CT images could be excluded by this manual procedure.

For the determination of root age of each segment at each time step, the reconstructed and stored root system of the precedent scan was opened simultaneously with the image of the subsequent scan. Using the overlay of both scans newly grown roots could be identified and added to the existing root structure. The temporal resolution of the growing root architecture was limited by the time interval between two CT scans (2 days). To obtain smoother root growth, the origination time *t_s_* of a segment *s* that grew between times *t_i_* and *t_i+_*_1_ when a CT scan was made, was calculated using Equation 2:

(2) $$t_{s}=t_{i}+\frac{l_{s}}{\Delta l_{s}}\left(t_{i+1}-t_{i}\right)$$

where Δ*l_s_* [L] is the length of all segments that grew between time *t_i_* [T] and *t*_*i* + 1_ and that are connected to the same connection point of the root system at time *t_i_* as the root segment *s*, and *l_s_* is the length of all segments that are closer to the connection point than segment *s* and therefore should have emerged before segment *s*. The average length of a manually reconstructed root segment was 0.087 ± 0.008 cm.

#### Destructive measurements

At the end of the experiment (Day 31–35) roots were extracted from the soil by washing using sieves of 3 and 2 mm mesh size successively. In the “Split” experiment, compartments were analyzed separately. In the “NoSplit” experiment, the roots grown into the lower 7 cm of the cylinder that could not be imaged were harvested separately. Roots were stored in Rotisol and subsequently scanned on a flatbed scanner (EPSON Perfection V700 PHOTO). The images were analyzed with WinRHIZO 2009b (Regent Instruments, Inc., Quebec, Canada) to obtain total root lengths.

#### Modeling of RWU

For the simulation of RWU we used the numerical model R-SWMS, which solves the water flow equation in the root network and in the soil (Javaux et al., 2008). The numerical solution of the Richards equation (Equation 3, Richards, 1931) with a sink term *S* based on SWMS_3D (Simunek et al., 1995).

The water flow equation for the root network is solved based on the radial and axial flow equations (**Equations 4 and 5**) and the mass balance at each root node, resulting in a system of linear equations for ψ*_x_*, the xylem water potential (Doussan et al., 1998). The system is solved with a biconjugated gradient method.

The root and the soil water flow equations are coupled through the definition of the sink term of the Richards equation and of the water potential at the soil-root interface for the Doussan equation. The sink term of the Richards equation is defined as the sum of the radial root flow into all root segments, *k*, located within a soil voxel (cuboid), *i*, divided by the cuboid volume (Equation 6). The soil-root interface water potential at each root node is defined as the distance weighted average of the water potential at the soil voxel nodes.

(3) $$\frac{\partial \theta }{\partial t}=\nabla \;\cdot \;\left[K\left(\psi \right)\nabla \left(\psi \right)\right]+\frac{\partial K\left(\psi \right)}{\partial z}+S(x,y,z,t)$$

(4) $$J_{r}=K_{r}^{*}A_{r}\left(\psi_{s,int}-\psi_{x}\right)$$

(5) $$J_{x}=-K_{x}^{*}A_{x}\;\left(\frac{d\psi_{x}}{dl}+\frac{dz}{dl}\right)$$

(6) $$S_{i}=\frac{\sum_{k\;=\;1}^{n_{k}}J_{r}^{k}}{V_{j}}$$

where θ [L^3^ L^−3^] is the volumetric water content of the soil, *K* [L T^−1^] the soil hydraulic conductivity, ψ [P] the soil matric potential, and *z* [P] the gravitational potential. *S* [L^3^ T^−1^] is the sink term, *J_r_* [L^3^ T^−1^] the radial flow into the roots, *J_x_* [L^3^ T^−1^] the axial flow in the root xylem, K^*r^ [L T^−1^ P^−1^] is the radial conductivity, K^*^_x_ [L^2^ T^−1^ P^−1^] the axial conductivity, ψ_*s*,*int*_ [P] is the water potential at the root-soil interface and ψ*_x_* [P] the xylem water potential, *A_r_* and *A_x_* [L^2^] are the lateral surface and the cross sectional areas of a root segment, *l* [L] is the length of a root segment. The axial conductance, *K_x_* = K^*^_x_A_x_ [L^4^ T^−1^ P^−1^]. The indices *i* and *k* stand for discrete soil voxels and root segments, respectively. *V_j_* [L^3^] is the volume of a single soil voxel.

The equivalent hydraulic conductivity of the root system, *K_root_* [L^3^ P^−1^ T^−1^], is defined by the relation between actual transpiration, *T_act_* [L^3^ T^−1^] and the difference between the effective soil water potential and the root collar potential (Javaux et al., 2013).

(7) $$T_{act}=K_{root}\left(\psi_{s,eff}-\psi_{collar}\right)$$

(8) $$\psi_{s,eff}=\sum_{j}SUF_{j}\;\psi_{s,int}$$

where ψ*_s,eff_* [P] is the effective soil water potential, which is weighted by the standard uptake fraction, *SUF_j_* [-]. *SUF_j_* represents the relative water uptake by a root segment *j* in a soil profile with a uniform soil water water potential and can be derived by solving the Doussan equations. A more detailed explanation can be found in Couvreur et al. (2012).

The R-SWMS code and a manual as well as the reconstructed root architectural files are available upon request from the authors.

#### Model setup

The samples NoSplit 2 from “NoSplit” experiment and Split 1 and Split 3 from “Split” experiment, with fully reconstructed root architectures, were used for the setup of virtual experiments in R-SWMS. In the following when referring to modeling data names of samples will be written in italics.

##### Soil domain

We defined rectangular domains with a discretization of 0.5 × 0.5 × 0.25 cm^3^. The domain size was 14 × 14 × 21.5 cm^3^ for the “NoSplit” experiment. The domains of the “Split” experiment differed in the z-direction (*z* = 20 cm for Split 1; *z* = 20.25 cm for Split 3, Figure 2B). The cylindrical geometry of the soil columns was approximated using Pythagoras' Theorem with a cylinder radius of 7 cm. Voxels belonging to this cylinder were defined as soil material; voxels on the outside were defined as wall material. The water retention characteristic was described by a bimodal Mualem - van Genuchten expression (Van Genuchten, 1980; Durner, 1994). The soil hydraulic parameters in Table 2 were derived from separate HyProp measurements (Peters and Durner, 2008), except the saturated hydraulic conductivity, *K_s_*, which was predicted using the Rosetta tool (Schaap et al., 2001). Paraffin layers were defined as 0.5 cm thick layers within the cylinder. The modeled layer thickness is thus 10 times larger than the thickness of the split layer in the experiment. However, to achieve a reasonable simulation speed, we had to settle for this trade-off. The split layer material was defined equal to the wall material. However, as a certain leakiness of the split layers became obvious during the time course of the experiment and later on during the modeling, we decided to simulate the leakage by assigning a small hydraulic conductivity to the layers of concern. All soil boundary conditions were defined as zero flux. Initial conditions were defined according to the initial water content at the start of the drying period in the experiments. In the “NoSplit” setup soil matric potential was at hydrostatic equilibrium and in the Split setup, soil water content was equal in each compartment.

**Table 2:** **Soil hydraulic parameters for the Mualem-van Genuchten expression**.

**Material**	**θ_r_ [cm^3^ cm^−3^]**	**θ _s_ [cm^3^ cm^−3^]**	**α [hPa^−1^]**	**n**	**w2**	**α2 [hPa^−1^]**	**n2**	**λ**	**K_s_ [cm d^−1^]**
Soil	0.01	0.35	0.05	4	0.35	0.0033	1.3	0.5	170
Wall	0.01	0.35	0.000003	1.5	–	–	–	0.5	0
Paraffin split/^*^semi	0.01	0.35	0.000003	1.5	–	–	–	0.5	0/0.001^*^

*Saturated and residual water content, θ_s_and θ_r_, respectively; van Genuchten shape parameters, α and n; pore connectivity parameter λ; and saturated hydraulic conductivity, K_s_. For the soil, a bimodal θ(ψ) relation (Durner, 1994) was used. Asterisk indicates the saturated hydraulic conductivity of paraffin for the scenario SC*.

##### Root architecture

**Figure 3:**
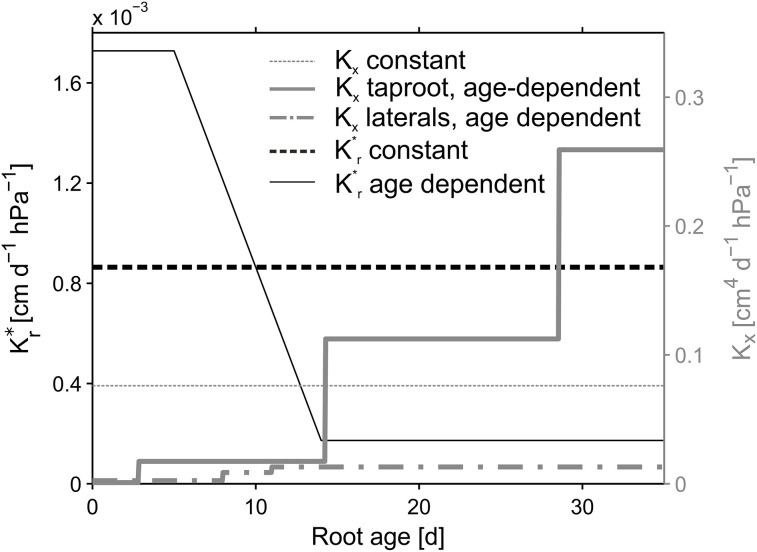
**Root hydraulic conductivities**. Reference parameterization is depicted in bold lines. Age dependent radial conductivity is equal for both, the taproot and laterals. Constant values were kept constant over root type and age.

The root architectures for the simulations were obtained from the manually reconstructed CT images. Root hydraulic properties were based on an age dependent parameter set by Doussan et al. (2006) for *Lupinus angustifolius* (Figure 3, bold lines). Radial conductivity of roots was given a constant value of 8.64 × 10^−4^cm d^−1^hPa^−1^. The axial conductances increased stepwise with segment age. In Doussan et al. (2006) axial conductance (i.e., xylem conductance) of lateral roots increased with age, whereas taproot axial conductance increased with distance to the tip. Thus, for the taproot we had to convert our age information to distance information. For this we divided the given distances by the mean measured elongation rate of the taproot (0.7 cm d^−1^) to translate the given distances to the according ages.

At a given simulation time only the root segments with an origination time smaller than the actual simulation time were taken into account. The root system was updated at each further run-time step thus enabling predefined root growth over time. We converted the measured daily transpiration rates of each sample to a periodic step function with zero flow during the night and so defined the root flow boundary conditions in the model at the root collar.

#### Scenarios

Each of the three samples was exposed to two or three scenarios to analyze the effect of paraffin layers on RWU. In the first scenario *(CD)*, a continuous soil domain without any split layers was used. In the second scenario *(NC)*, we defined three non-conductive paraffin layers. Finally, the third scenario *(SC)*, aimed to achieve best agreement to measured data for the “Split” experiment by considering leaking paraffin layers and assigning a low hydraulic conductivity of 0.001 cm d^−1^ (Table 2) to the split layers. Sample Split 1 was simulated with three slightly conductive layers, and Split 3 with a non-conductive layer at −5 cm and two remaining slightly conductive layers.

A sensitivity analysis was performed to evaluate the uncertainties in the modeling approach due to uncertain age dependent root hydraulic conductivities. We focus on predawn water potentials, ψ*_d_*, since simulated soil water potentials could be compared with measurements and transpiration rates were used as boundary conditions. Equation 7 shows that in case of zero transpiration, e.g., during night, ψ_*s*,*eff*_ = ψ_*collar*_. Thus, predawn water potential is independent of *K_root_* and *SUF* can be used as an indicator for the impact of different root hydraulic conductivities on ψ*_pd_*. Since *SUF* represents the water uptake by a root segment, relative to the total of the uptake of the root system, *SUF* does not depend on the absolute (radial and axial) conductivities of the root segment but on the ratios between the conductivities of one segment to other segments.

The variability of *SUF* induced by different age dependencies of the hydraulic parameters was examined by comparing different combinations of age dependent and constant axial and radial conductivities for the different reconstructed root architectures (NoSplit2, Split1, Split3) at the end of the growth period. The constant value for *K_x_* was defined as the arithmetic mean of the age dependent *K_x_* values and age-dependent K^*^_r_ values were modified from Doussan et al. (1998) who defined age-dependent K^* r^ values for *Zea mays L*. (Figure 3). An overview of the parameterization is given in Table 3.

**Table 3:** **Perturbations of root hydraulic conductivities from Figure 3 for the sensitivity analysis**.

	K^*^_r_	***K_x_***
Reference	Constant	Age dependent
1	Constant	Constant
2	Age dependent	Constant
3	Age dependent	Age dependent

## Results

### Experimental results

**Figure 4:**
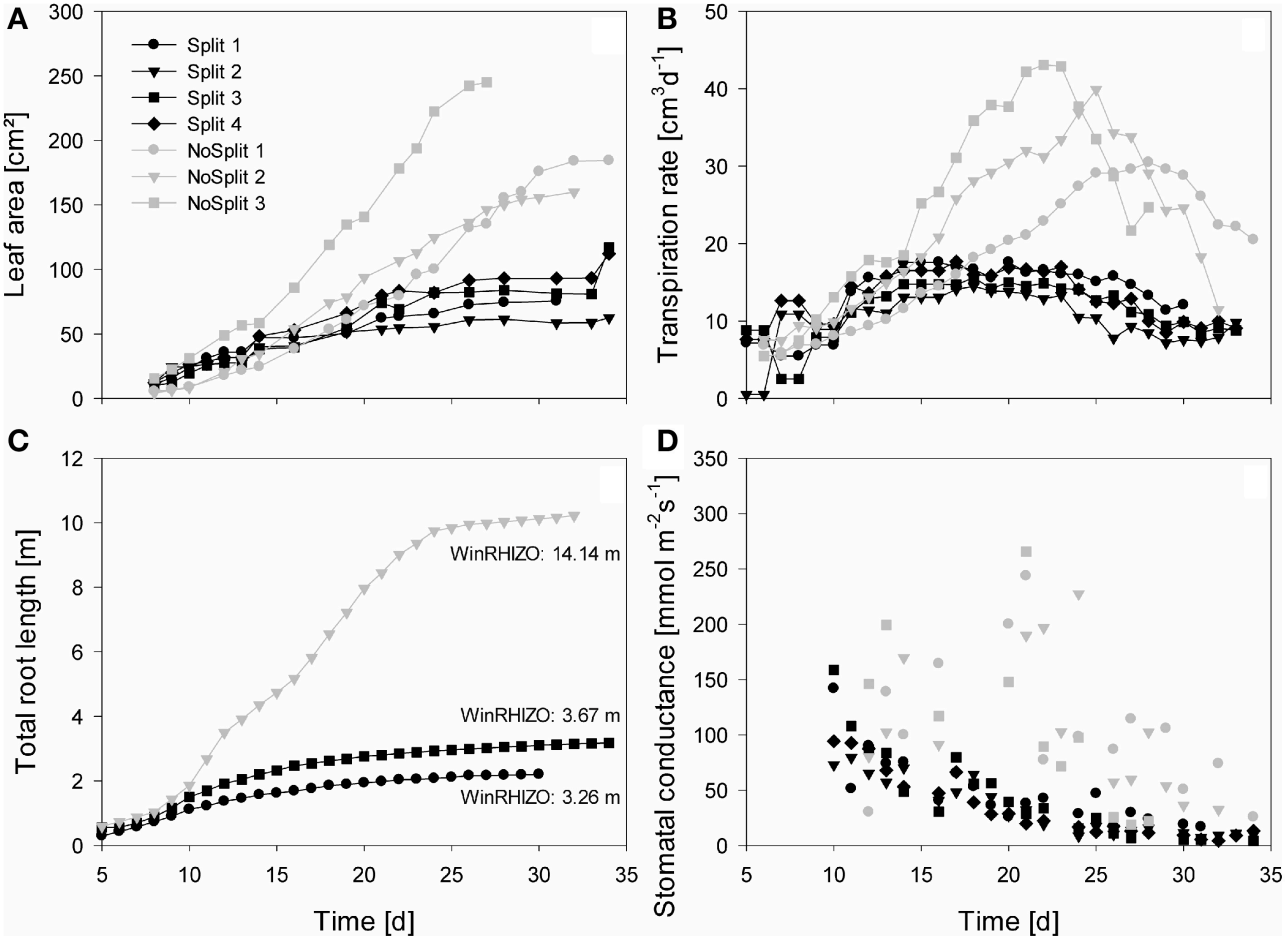
**Measured plant traits over time from Day 5/10 until Day 35**. Gray symbols represent the NoSplit setup and black symbols the Split setup. Different symbols represent replications. **(A)** One-sided leaf area, **(B)** Transpiration rate, **(C)** total estimated root length of the samples used for modeling, **(D)** stomatal conductance of the youngest unfolded leaves, data points represent the mean of two measurements.

As expected, plant performance differed markedly between the two experiments (Figure 4). In the “NoSplit” experiment plants were bigger and had a larger leaf area (Figure 4A). Leaf growth was initially the same in both experiments, but after Day 15 leaf area increased more in the “NoSplit” experiment. A similar pattern could be observed for total root lengths obtained from CT images over time (Figure 4C). Root elongation was similar for both, “Split” and “NoSplit” experiment until Day 10. Afterwards elongation rate was higher for “NoSplit.” Root length estimations from destructively harvested roots using WinRHIZO were on average higher than estimations from CT (Table 4).

**Table 4:** **Root length estimations from CT images and from destructive measurements at the end of each experiment**.

		**Length CT [cm]**	**Length WinRhizo [cm]**	**(WinRhizo -CT)/(WinRhizo [–]**
NoSplit 1		–	1504	–
NoSplit 2		1022	1414	0.27
NoSplit 3		–	2023	–
Split 1	Total	270	326	0.17
	Comp. I	196	240	0.18
	Comp. II	44	48	0.08
	Comp. III	20	27	0.26
	Comp. IV	10	11	0.10
Split 2	Total	–	335	–
	Comp. I	–	79	–
	Comp. II	–	213^*^	–
	Comp. III	–		–
	Comp. IV	–	43	–
Split 3	Total	319	368	0.13
	Comp. I	126	132	0.05
	Comp. II	64	69	0.07
	Comp. III	90	125	0.28
	Comp. IV	38	41	0.07
Split 4	Total	–	573	–
	Comp. I	–	143	–
	Comp. II	–	234	–
	Comp. III	–	158	–
	Comp. IV	–	38	–

* *Value for Compartments II and III combined*.

The vertical root length distribution in the “Split” experiment differed between Split 1 and the remaining samples. Compartment I in Split 1 contained about 3/4 of the total root length, while the distribution for the other replications of the “Split” experiment was more even (Table 4). In the “NoSplit” experiment root density increased with depth.

In both experiments transpiration rate initially increased with leaf area (Figure 4B). In “NoSplit” a sharp decrease in transpiration rate was seen at Days 23, 25, and 28, respectively for the different samples. Transpiration reduction occurred earliest in NoSplit 3, which was also the largest plant with the highest transpiration rate up to that day. In the “Split” experiment, transpiration reduction could be observed earlier, although the reduction in transpiration was not as strong as in the “NoSplit” experiments. The lower leaf areas and smaller transpiration rates in the “Split” experiment were accompanied by lower stomatal conductance of the youngest unfolded leaves in comparison to the “NoSplit” experiments (Figure 4D). Stomatal conductance decreased already from the first measurement, i.e., Day 10, in the “Split” experiment. In the “NoSplit” experiment the variability of stomatal conductance in the different samples was very high, but low values were not measured until Days 23 or 24, respectively.

**Figure 5:**
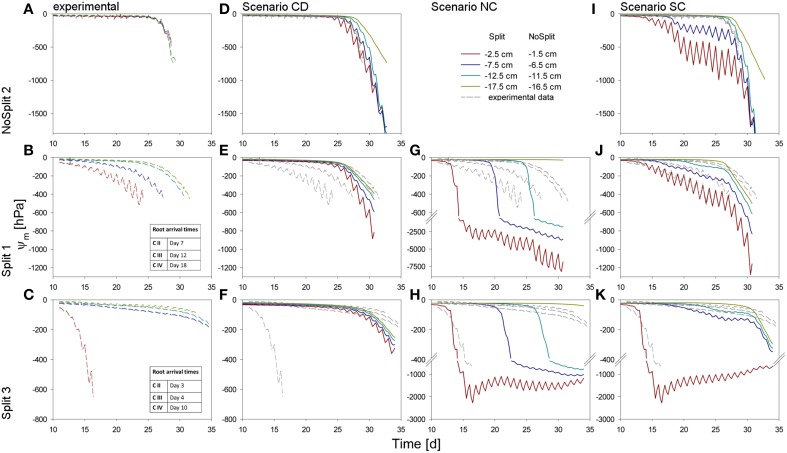
**Soil matric potentials for the three samples (top to bottom) within the different compartments. (A–C)** Values measured by the tensiometers in the experiments. **(D–K)** Comparison of different scenarios with the measured values, repeated in dashed, gray lines. **(D–F)** Simulation CD – unrestricted, continuous soil domain, **(G,H)** Simulation NC – impermeable, non-conductive layers, **(I–K)** Simulation SC – semi-conductive layers.

The addition of paraffin layers (“Split” experiment) also had a pronounced effect on the temporal development of the soil matric potentials in the different soil compartments (Figures 5A–C). For the sake of brevity we only present the results of the samples that were later used for modeling (the remaining samples behaved similarly, see Supplementary Figure 1). In NoSplit 2, soil matric potential remained high during a long period (approximately until 25 days after the start of the experiment) and there were only small differences between the matric potentials at different depths. After 25 days, the time at which the transpiration in the no-split experiment started to decrease (Figure 4B), the matric potentials decreased strongly and more or less simultaneously at different depths in the column. For the “Split” experiments, the matric potentials started to decrease much earlier (from Day 10 onwards) and sequentially from the top toward the bottom compartments. Except for the upper compartment in Split 3, the decrease of matric potential was more gradual and less abrupt than in the “NoSplit” experiments. The tensiometer readings for the “Split” experiment showed a pronounced day-night cycle in the upper and a more damped diurnal signal in the lower compartments.

**Figure 6:**
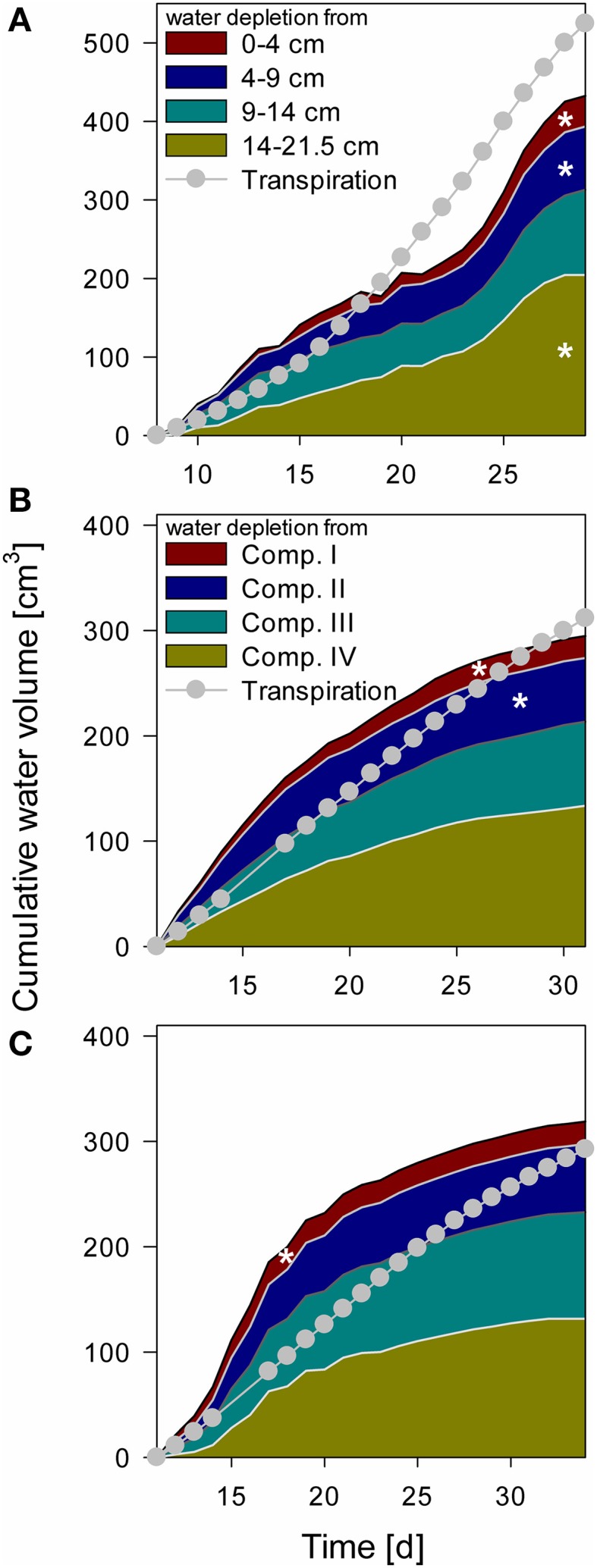
**Cumulative water depletion from each compartment over time compared to cumulative transpiration from Day 8 for NoSplit (A) and Day 11 for Split 1 (B) and Split 3 (C) until the end of the experiment**. Filled areas represent cumulative water content change in the different compartments calculated from tensiometer measurements. Gray line and circles represent cumulative transpiration measured with balances. White asterisks denote the point, when the tensiometer in the compartment showed air bubbles.

Water depletion from each compartment was calculated from measured tensiometer values assuming a uniform matric potential within a layer and using the substrate specific water retention curve (Table 2). These data were compared to total water loss derived from weighing cells (Figure 6). When air bubbles started to form in the tensiometers no further water content change could be calculated. The calculated water content at this point was between 9.5 and 10.6% (ψ_m_ = −745 to −431 hPa). In the “NoSplit” setup (Figure 6A) there were no true compartments, we therefore assumed that the tensiometers represented the matric potential for the surrounding volume closest to the tensiometer. While the difference between calculated and measured cumulative water depletion for the “Split” setup (Figures 6B,C) converged to below 10% (+9% Split 3, −5% Split 1) at the end of the experiment, it was much higher (17%) in the “NoSplit” setup. Comparison of the slopes over time indicates a poor fit of the dynamics. Calculated water depletion was clearly overestimated at the beginning and underestimated toward the end of the experiments, especially in Split 3.

The arrival of roots in Compartments III and IV in Split 1 was at Day 12 and 18, respectively, nonetheless there was significant (even if overestimated) water depletion from both compartments before these dates.

### Simulation results

The three samples (NoSplit 2, Split 1, and Split 3) representing different RSA were subjected to three different scenarios: (*CD*), a continuous, unrestricted soil domain, (*NC*) a soil domain with non-conductive split layers, and (*SC*) with semi-conductive split layers. Mean simulated soil matric potentials in four layers were compared to the measured tensiometer values (Figure 5).

#### Choice of scenario

In scenario *(CD)* (continuous soil domain) (Figures 5D–F), the simulated matric potentials in the different soil layers started declining strongly and nearly simultaneously only toward the end of the simulation period. The simulated decline occurred the earliest and was the strongest in the “NoSplit” experiment reflecting the larger cumulative transpiration from this experiment.

For the “NoSplit” experiment, the simulated matric potentials for scenario *(CD)* showed a similar behavior as the measurements (Figure 5D). The timing and the slope of decrease fitted the experimental data well. The lowest tensiometer (−16.5 cm) was an exception, probably due to the fact that the deep roots could not be detected in the CT and were missing in the model.

For both samples of the “Split” experiment (Figures 5E,F), the measured matric potentials of the upper two tensiometers started decreasing much earlier than the simulated matric potentials for scenario *(CD)*. This illustrates the effect of the paraffin layers on the soil water distribution in the “Split” experiment which is ignored in scenario *(CD)*.

Scenario *(NC)* with non-conductive paraffin layers was simulated only for the “Split” experiments (Figures 5G,H). The simulated matric potentials at the tensiometer depths decreased sequentially from top to bottom and the time lag between these decreases was much larger than in scenario *(CD)* for the same samples. The simulated water potentials started to decrease shortly after roots arrived in a compartment. In Split 3 (Figure 5H), simulated average water potential in Compartment I decreased to about −2000 hPa until Day 15 and remained at this level thereafter only showing pronounced diurnal fluctuations until the end of the simulation run. In both samples of the “Split” experiment (Figures 5G,H) for scenario *(NC)* the simulated changes in water potential in Compartment IV were very small due to the small fraction of roots in this compartment.

With Scenario *(NC)* we were not able to reproduce the measured dynamics of soil matric potentials of the “Split” samples. Measured matric potentials did not show a sequential stepwise decrease but a more gradual decrease that started earlier than the simulated decrease and sometimes even earlier than the root arrival time in a compartment. One exception was the matric potential in Compartment I of the Split 3 sample. Scenario *(NC)* produced large water potential differences between the different compartments, which were not in agreement with the measurements.

The previously described results indicate that paraffin layers were not perfectly isolating, but that there must have been water redistribution between neighboring compartments, albeit at a lower rate than in completely unrestricted soil. Thus, scenario (*SC*) was applied.

For Sample Split 1 in scenario *(SC)* (Figure 5J), the simulated matric potentials of Compartment I showed a slower decrease than those obtained with scenario *(NC)* or *(CD)*. At the same time scenario *(SC)* resulted in an earlier decrease of matric potential in the lowest compartment compared to scenario *(NC)*. The pronounced measured diurnal pattern of soil matric potential in Compartment I was successfully reproduced in scenario *(SC)*.

Likewise, for Sample Split 3 simulated matric potentials of scenario *(SC)* showed the best agreement with measured tensiometer data. Here the assumption that all layers except the top layer were leaking was important for obtaining the good agreement.

As expected, for the “NoSplit” experiment (Figure 5I), agreement between measured soil matric potentials and those simulated with scenario *(SC)* was very poor. However, it is interesting to note the influence of, albeit leaking, hydraulic barriers to soil water potentials.

In contrast to experimental approaches, which can only detect changes in soil matric potential, the simulation results allow disentangling the different fluxes which contribute to local changes in matric potential and soil water content. The evaluation of fluxes was restricted to those simulations which showed the best agreement between measured matric potentials and simulated once, i.e., scenario *(CD)* for sample *NoSplit 2*, scenario *(SC)* for samples *Split 1* and *Split 3*.

#### Simulated flow dynamics

**Figure 7:**
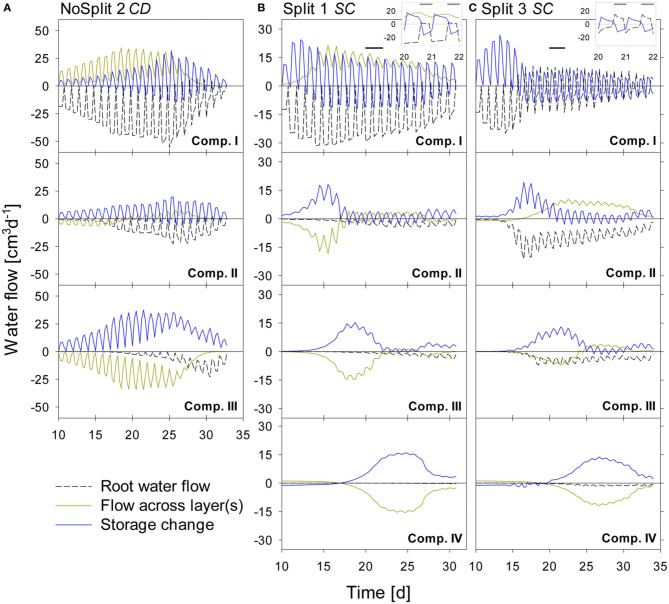
**Modeled water flow dynamics over time in the (A) NoSplit *CD*, (B) Split1 *SC*, and (C) Split 3 *SC* scenarios**. Dashed black lines represent root water flow. Dark yellow lines represent the net flow across the paraffin layers from neighboring compartments. Negative values indicate water removal, positive values water addition to a compartment, respectively. Blue lines represent the resulting change of soil water content in the compartment with positive values denoting a decrease in water storage and negative values and increase in storage. Plotted values are flow rates at four discrete times per day. Because there is only one value for the night phase, flows at night appear as single peaks. The inlays at the top show the dynamics in Compartment I between Days 20–22 (as indicated by the black bars) at a higher temporal resolution (10/d), showing the dynamics of RWU and hydraulic redistribution.

The water balances of the single soil compartments are depicted in Figure 7. In case of impermeable split layers, the storage change within one soil compartment should equal root water uptake. However, if the split layers are leaking, which is the case for most of the layers, only adding the net flow through the split layers to the storage change equals root water uptake.

For the *NoSplit 2* (Figure 7A) simulation RWU was largest in the upper compartment, where it started to decrease from Day 25 onward. The 5–10 cm layer only started to significantly contribute to RWU from Day 17 onward and the 10–20 cm layer only after Day 20, which is related to root arrival time.

It is interesting to note that “early morning values” of RWU in the 0–5 cm layer remained higher than those in the other layers even after 25 days, i.e., during a period where overall contribution of the lower layers to RWU had increased and total transpiration rate was reduced in the experiment.

Simulations showed soil hydraulic redistribution of water from the lower layers to the top 0–5 cm. At 5–10 cm depth inflows from the deepest soil layer and outflows to the 0–5 cm layer were almost of the same magnitude, so the resulting net flow oscillated around zero. Soil hydraulic redistribution started to decrease after Day 25 and seized after Day 31.

Since RWU from a layer corresponds to the sum of the net water flow into and the decrease of the water storage in a soil compartment, it is evident that RWU in a soil layer cannot be derived from water storage changes in that layer. RWU in the 0–5 cm layer is considerably larger than the changes in water storage whereas the opposite is true for the 10–15 cm layer. It is clearly visible that RWU and storage change did not correspond to each other as long as there was significant soil hydraulic redistribution.

Substantial soil hydraulic redistribution occurred also in the samples *Split 1* (*SC*) and *Split 3* (*SC*), although *K_s_* values of paraffin layers were only 0.001 cm d^−1^ (Figures 7B,C). In both simulations RWU did not correspond to water storage change with the exception of Compartment I in *Split 3*, which was assumed to be separated by a non-conductive split layer. RWU from Compartment IV was very small in both *Split 1* (*SC*) and *Split 3* (*SC*) while the change in soil water content was substantially higher due to flow across the split layer. The same pattern was observed in Compartment III, but net outflow of water started earlier and was eventually compensated by inflow from Compartment IV. Compartment II showed a contrasting behavior between the two samples of the “Split” experiment. In *Split 3* the non-conductive layer at the top prevented water movement in the soil to Compartment I, and the fraction of RWU from compartment II was considerably higher in *Split 3* than in *Split 1*.

In both simulations of the “Split” experiment, there was significant hydraulic redistribution via deep roots into Compartment I. Root hydraulic redistribution was much more pronounced in *Split 3*. According to the simulations the redistribution via the roots occurred during night and the water was taken up by the roots during the next day.

The comparison of cumulative root water uptake from the different compartments with cumulative water depletion at the end of the simulations highlights the importance of including soil hydraulic redistribution when analyzing the pattern of RWU (Table 5). This is most obvious in the unrestricted sample *NoSplit 2*, where 69% of RWU occurred in the 0–5 cm layer, while the water depletion in this layer was only 16% of total water depletion. But even in Compartment I of *Split 3*, which was assumed to be perfectly isolated, RWU and water depletion are slightly different, which is probably due to the discretization of simulation outputs and rounding errors.

**Table 5:** **Total root water uptake and water depletion in each soil compartment at the end of each simulation**.

**Simulation**		**RWU [cm^3^]**	**Water depletion [cm^3^]**
*NoSplit 2 CD*	Total	660.4	657.4
	Comp. I	456.2	105.8
	Comp. II	124.3	139.6
	Comp. III	79.9	412.0
*Split 1 SC*	Total	387.7	386.7
	Comp. I	336.8	121.6
	Comp. II	32.2	82.4
	Comp. III	17.2	84.6
	Comp. IV	1.5	98.2
*Split 3 SC*	Total	358.4	358.2
	Comp. I	101.8	97.8
	Comp. II	175.5	87.8
	Comp. III	66.7	81.9
	Comp. IV	14.4	90.6

Further, the development of the root system architecture (Figure 2) can be compared to the water flows within the soil and root system (Figure 7). Due to the semipermeable split layers in *Split 1*, most of the RWU takes place in the upmost compartment, the location where also most of the roots are found. In *Split 3*, where the top compartment is hydraulically isolated, the roots take up most of the water from this layer within the first 15 days, while afterwards the uptake shifts to the lower compartments. This pattern is reflected in the RSA development. The *NoSplit* setup shows a more or less smooth shift of roots as well as RWU downward in the domain.

#### Sensitivity analysis

**Figure 8:**
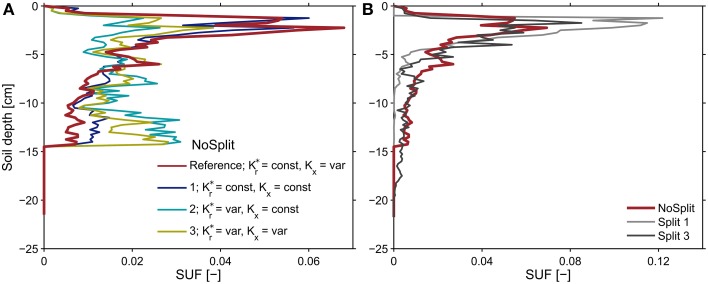
**Sums of the standard uptake fraction over soil depth increments of 0.25 cm for (A) the NoSplit root system at *t* = 32 days solved for different parameterizations of radial and axial root hydraulic conductivities and (B) for the reference parameterization of root hydraulic conductivities for the three different plant architectures**. The observed variability for the two split setups was less than shown in subplot **(A)** and is shown in Supplementary Figure 2.

Following Equation (8), the effective soil water potential, in case that transpiration is zero, is equal to the water potential at the root soil interface weighed by the standard uptake fraction, *SUF*. The *SUF* was calculated for four different parameterizations of root hydraulic conductivity. Figure 8A shows the sum of *SUF* for the NoSplit setup within given soil depth increments. With age-dependent radial conductivity the *SUF* becomes more uniform over depth. For both Split setups the variability with the different parameterizations is not as large (see Supplementary Figure 2).

The SUF, which shows the hydraulic architecture of the root systems, are compared for the three different plants (Figure 8B). In contrast to the root system architecture, only small differences can be observed. The differences in predawn water potentials between the different plants were thus mainly due to the soil water distribution and less to RSA.

#### Pre-dawn water potential at the root collar

**Figure 9:**
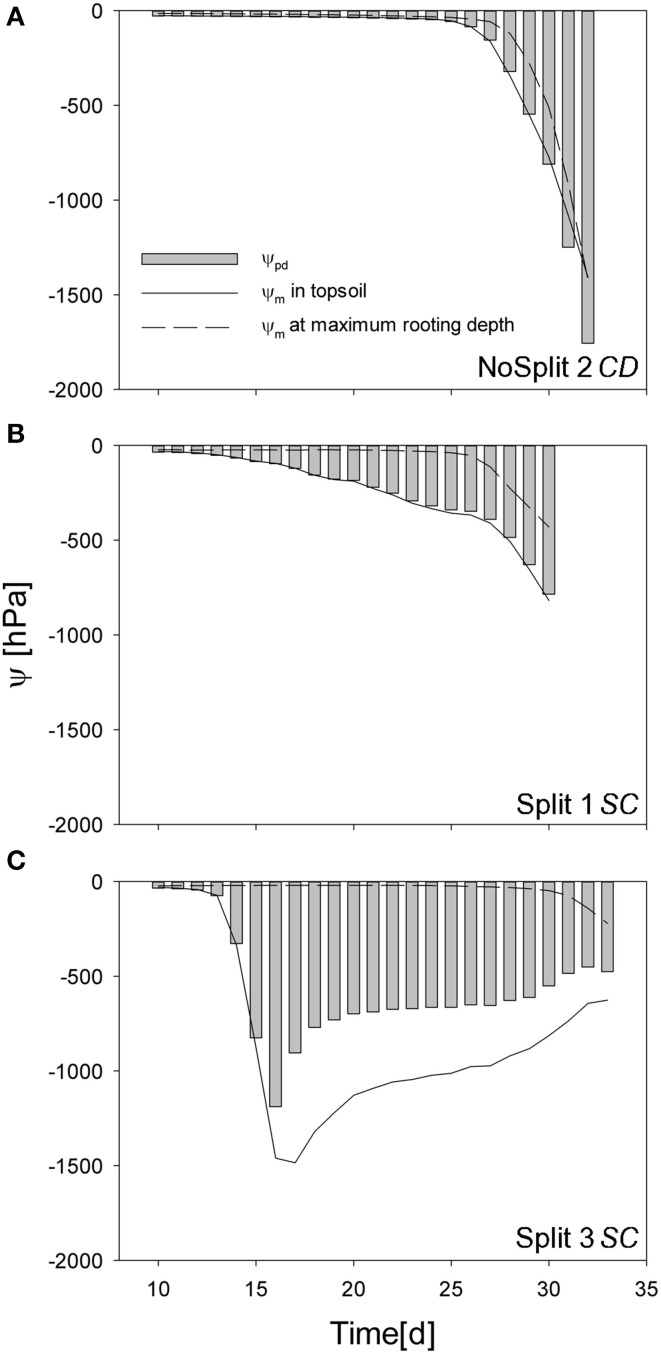
**Simulated predawn water potential at the root collar (ψ_collar_ gray bars) for (A) NoSplit 2 *CD*, (B) Split 1 *SC*, and (C) Split 3 *SC* and simulated soil water potentials (ψ_m_) at the top 5 cm depth (full line) and at the maximum rooting depth (dashed line) over time**.

Simulated pre-dawn water potential at the root collar (ψ*_pd_*) was used as an indicator for plant water status (Figure 9). ψ*_pd_* is independent of actual transpiration rates and can therefore be used to compare different samples. ψ*_pd_* is generally thought to be in equilibrium with the soil water potential provided that night induced interruption of transpiration is long enough and flow rates in soil root systems are high enough to reach this equilibrium (Donovan et al., 2003). However, the soil matric potentials, simulated in this study were clearly not in equilibrium, especially for the two split samples.

In sample *NoSplit 2 (CD)*, simulated predawn ψ*_pd_* decreased only slowly until Day 25 and was in equilibrium with soil matric potential in the topsoil (−1.5 cm depth). Due to the homogeneous soil water distribution it was also closely related to the matric potential in the wettest soil accessible to the plant, i.e., the soil at maximum rooting depth at each time step. From Day 25 onwards there was a strong decrease of soil matric potential in the whole column and an according decrease of ψ*_pd_*. After Day 30, ψ*_pd_* was more negative than the topsoil matric potential. The disequilibrium increased until the end of the experiment. In both split samples ψ*_pd_* was more negative than the matric potential at maximum rooting depth but less negative than the topsoil matric potential, indicating that the system did not reach equilibrium at the end of the night. ψ*_pd_* in *Split 1 (SC)* was closer to the matric potential in the topsoil, reflecting the higher redistribution through the split layers in *Split 1 (SC)*.

**Figure 10:**
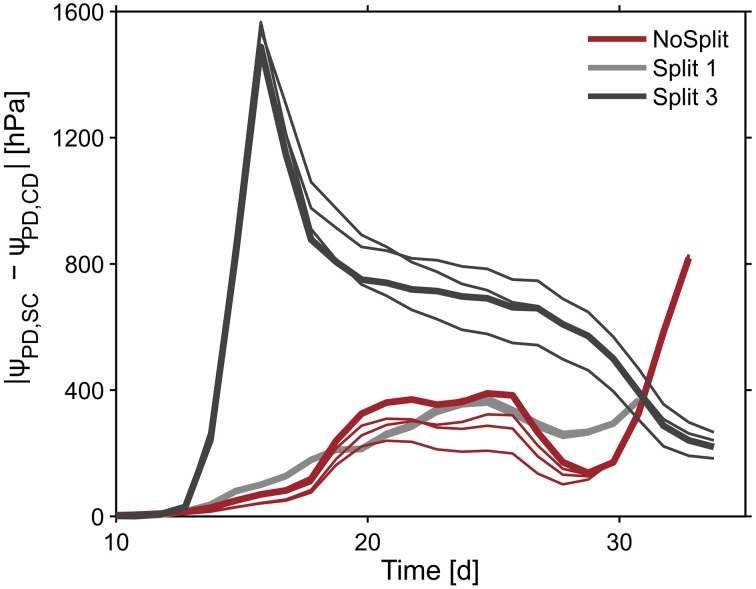
**Influence of split layers on simulated soil water potentials for the reference parameterization (bold lines) and for the remaining three parameter sets for root hydraulic conductivities (thin lines, Table 3)**. The soil water potential was calculated based on scenarios for uniform distribution of soil water potential (Equation 8). The four lines overlap in the Split 1 setup.

To illustrate the impact of the split layers on soil and thus plant water status, predawn soil water potentials of the different scenarios with and without paraffin layers (*SC* vs. *CD*) for each sample were compared. The difference of absolute soil water potentials for the two contrasting soil environments was calculated (Δ/ψ*_pd_*/ = /ψ*_pd_*/ _SC_ − /ψ*_pd_*/ *_CD_*) (Figure 10, bold lines). As expected, soil water potential was constantly more negative in scenario *SC* than in scenario *NC*. Δ ψ*_pd_* in *Split 1* and in *NoSplit 2* were of the same magnitude, while in *Split 3*, where the upper paraffin layer was assumed to be non-conductive, it increased more rapidly and stronger, indicating an effect of the higher degree of hydraulic isolation of the different soil layers.

When using the previously calculated *SUF* to determine the impact of parameterization of root hydraulic conductivities on effective soil water potentials, the variability of soil water potentials compared to the plant variability is very small (Figure 10, thin lines).

## Discussion

### Influence of paraffin layers on plant growth

CT measurements gave insight into the changes of growth behavior caused by the addition of wax layers. However, the causes for these changes are not revealed by the CT measurements. By using a simulation model CT measured RSA and the low (zero) hydraulic conductivity of the wax layer could be linked to internal plant water potentials. This enables an interpretation of plant water stress and its implications for shoot and root growth.

Experimental results as well as simulations suggested strongly that most of the paraffin layers were not perfectly hydraulically isolating. Tomographic images and visual inspection after destructive harvest showed, however, no evidence of cracks or holes in the wax layers. It is possible that there were cracks at the container walls that were formed due to shearing of the paraffin caused by the weight of the soil in the upper compartments. The only paraffin layer that was evidently tight was consequently the uppermost layer in the sample Split 3. Drew (1975) suggested the use of layers as thin as 0.2 mm, which is even thinner than the layers that were used in this study. Another possible source of leakage is linked to diurnal shrinking and swelling of roots (Huck et al., 1970), which could lead to cavities in the paraffin where it is penetrated by roots. This could not be excluded as CT images were recorded during night.

Roots easily penetrated the paraffin and grew into the lower compartments. Taproots and vertically oriented laterals were not affected by paraffin layer. However, a few roots continued to grow horizontally within the soft paraffin layers (see Supplementary Figure 3).

The plants in the “Split” experiment were overall smaller with lower root densities. Inserting split layers generated a substantial resistance to vertical water flow within the soil and hence water redistribution in the soil column. A restriction of this redistribution led to lower simulated predawn root and collar water potentials, which were related to lower measured stomatal conductance. The lower predawn water potentials pointed at plant stress that resulted in a restriction of root and shoot growth. Even though the root-shoot ratio was shown to increase in *Vicia faba* in drier environments (El Nadi et al., 1969), this could not be observed in this experiment. A possible explanation for this is the higher bulk density in the split experiment. Slight increases of soil strength can lead to a substantial reduction of root penetration rate (Taylor et al., 1966). We cannot exclude a possible effect of oxygen depletion on plant performance caused by the addition of paraffin layers, as no oxygen concentrations were measured. However, we feel that hypoxia is highly unlikely: The soil was initially not water saturated and the fact that paraffin layers were permeable to water means that soil air could move as well. The rhizon samplers were kept in the soil during the experiment as possible pathways for air. The soil mixture was an artificial mixture without added organic matter, so microbial respiration should be minimal. Experiments with the same quartz substrate showed that even close to saturation redox potentials only decreased after adding significant amounts of organic material (Ackermann et al., 2008).

### Relation between measured water loss and RWU

The simulations showed the discrepancy between change in soil water content and the location of root water uptake for individual soil compartments, which was caused by soil hydraulic redistribution. Even a small conductivity of the hydraulic barriers led to considerable redistribution of soil water. The direct calculation of soil water content, and in extension RWU, from measured soil matric potentials was further complicated by the non-linear relation between water potential and water content, which precludes the extrapolation of a single tensiometer reading to the total soil compartment without knowing the gradients. The development of gradients around active roots is shown in Supplementary Figure 4.

Even if the vertical soil flow is completely restricted, hydraulic redistribution through the roots might still be a substantial amount of water that is exchanged between the roots and the soil in the drier regions of the root zone. In this case, however, the net water content change should correspond to net root water flow. The share of root hydraulic redistribution was higher when soil water redistribution was restricted by barriers, allowing the formation of a sufficing water potential gradient to drive flow. This may in part explain the controversy in literature as to the ecological relevance of root hydraulic redistribution. Its magnitude spans almost two orders of magnitude and is affected by numerous factors, such as root and water distribution, soil texture, and root-soil hydraulic conductance (Neumann and Cardon, 2012).

### Predawn collar potential

Simulation results suggest that predawn collar water potential (ψ*_pd_*) cannot be related to the water potential in the wettest part of the root zone, as was previously reported in literature (Hinckley and Bruckerhoff, 1975) (Figure 9). When gradients in soil water potential increase ψ*_pd_* is closer to the driest part of the root zone as water redistribution in the soil is restricted by low unsaturated hydraulic conductivity. Disequilibrium between plant and soil water potentials was caused by the heterogeneity of soil water potential, as previously experimentally shown by Améglio et al. (1999) and Donovan et al. (2003). Root hydraulic redistribution can contribute to the disequilibrium as the nocturnal water loss prevents the recovery of plant water potential (Donovan et al., 2003). This leads potentially to the equilibration of the system but is ultimately limited by the soil-root resistance to water flow. The largest redistribution in the model, however, takes place through the leaking split layers (Figure 7). For this reason, in *Split 1 (SC)*, where the leakage caused the deeper layers to dry earlier, ψ*_pd_* was very close to the potential of the dry topsoil, while in *Split 3 (SC)*, with Compartment I being perfectly hydraulically isolated, ψ*_pd_* was between the potentials of the topsoil and the soil at maximum rooting depth.

### Determination of RSA with CT

Comparison of destructive WinRhizo scans and CT imaging showed a discrepancy of up to 27% for total root length between both methods (Table 4). Underestimation of root length with CT imaging had several reasons: (i) 3.5% of total root length had diameters <0.5 mm. As a diameter of twice the resolution (voxel side length 245 and 277 μm, respectively) is required for a safe detection, these roots were possibly missed by CT imaging (Koebernick et al., 2014). (ii) Roots that grow along the cylinder walls are often lost in the course of data processing, when edges of the domain have to be removed. (iii) In the “Split” setup, roots sometimes remained within the soft paraffin layers. These were eventually undetectable with X-ray CT as there is no density contrast between paraffin and roots. (iv) A possible effect of the changing soil moisture content on the segmentation cannot be excluded, since destructive measurements were only available for dry conditions at the end of the experiment. Especially at high soil moisture contents the segmentation of roots can be increasingly difficult (Flavel et al., 2012; Zappala et al., 2013). Conversely, Lontoc-Roy et al. (2006) had more difficulties segmenting maize roots from loamy sand under dry than under water saturated conditions. Our temporally repeated X-ray CT scans suggests that, for the relatively coarse roots of *Vicia faba* (mean diameter = 1.06 mm), water content did not strongly affect the segmentation results until the end of the experiment, when soil cracks started to form in the upper compartment of Split 1, which prevented the successful segmentation of nearby roots (Figure 2A).

### Parameterization of root hydraulic conductivity

Information on root hydraulic conductivities is very sparse. The use of the xylem pressure probe to determine axial and radial root hydraulic conductivities is technically very demanding, particularly for soil grown plants. Most applications refer to solution culture studies. The root hydraulic parameters for this study were derived from literature data based on experiments with lupin plants (Doussan et al., 2006) and could not be validated by direct measurements or simulation results. Thus, these parameters are a major source of uncertainty. So far, three major uncertainties could be identified:

1. The absolute value of the conductance of the root system, *K_root_*, and how it differs between plants
  This would affect the absolute value of simulated collar water potentials when transpiration takes place but it does not affect the predawn water potential. Thus, the conclusion that split layers reduce the collar pre-dawn water potential compared to a case where there are no split layers is not affected. The distribution of the water uptake when the soil water potential is non-uniform in the soil profile is affected by uncertainty in the absolute conductance of the root system. However, the relatively good agreement between simulated and measured soil water potentials indicates that the distribution of the root water uptake was simulated satisfactorily using the chosen root conductivities.
2. The ratio between *K_r_/K_x_*
  Previous simulation studies have shown that this ratio affects the location of root water uptake (Couvreur et al., 2014). When *K_r_/K_x_* is small, root water uptake occurs more uniformly along the root profile, whereas for higher *K_r_/K_x_* root water uptake occurs closer to the root collar. In this study we have additional root growth, which affects the location of water uptake. Again, the relatively good predictions of the soil water potentials indicate that the root water uptake distribution was simulated quite accurately.
3. The change of K^*^_r_ and K*_x_* over root segment age
  A sensitivity analysis showed that uncertainty about the age-dependency of the root hydraulic parameters has only a small influence on the predawn water potential. However, the age dependency affects the development of the hydraulic conductivity of the total root system and hence also the xylem water potential during transpiration. Further, the root hydraulic properties used in the model could be validated and/or optimized by additional measurements of water potential in the collar or the leaves. The most reliable measurement of leaf water potential (pressure chamber, Scholander et al., 1965) is destructive and hence not suitable for measurement of changes over time. Lately developed sensors for leaf turgor (ZIM-probes, Zimmermann et al., 2013) have the potential to overcome this problem. However, for given root architecture and transpiration rates, the ranking of the collar water potentials that were simulated for our experiments will remain the same if the hydraulic properties of root segments and their dependency on age are assumed to be the same for all plants.

## Conclusion and outlook

The initial goal was to disentangle root water uptake dynamics in a soil environment with strong water potential gradients. We addressed this question using a novel approach combining experiments, CT scanning and a simulation model. Notwithstanding the uncertainties that arise due to parameterization of the model we demonstrated the synergisms that emerge from combining split root experiments with model simulations and came to the following conclusions:

1. In horizontal split experiments not only the soil hydraulic redistribution is altered, but whole plant performance.
2. Using a simulation model in combination with data of the root architecture development, we found that the split layers generated an important resistance to vertical water flow or water redistribution in the soil column. Vertical redistribution of water was an important process to provide the root system with sufficient water for uptake. A restriction of this redistribution led to lower simulated predawn root and collar water potentials which were related to lower measured stomatal conductance. The lower predawn water potentials pointed at plant stress that resulted in a restriction of root and shoot growth.
3. Vertical redistribution along water potential gradients in the soil makes it generally impossible to link local root water uptake with local changes in soil water content. Also in split root experiments, which are designed to reduce this redistribution, redistribution might nevertheless be important when large differences in soil water potentials between compartments emerge despite low hydraulic conductivities of split layers.
4. If vertical redistribution of water through the soil is restricted, there may be nevertheless a substantial amount of water that is exchanged between the roots and the soil in the drier regions of the root zone.
5. Simulation results suggest that predawn collar water potential can only be related to the wettest soil water potential in case of low heterogeneity. In case of soil moisture heterogeneity the predawn water potential is closer to the dry soil part.
6. Paraffin layers are not perfectly hydraulically isolating different soil compartments.
7. Conclusions 2–6 could not have been made without soil and root water flow simulations. To setup the model, data on the dynamic root architecture was essential. The agreement between measured and simulated soil water potentials and their dynamics for the different root architectures and experimental conditions (scenarios for the different soil setup) while making use of the same set of root hydraulic and soil parameters for all the simulated experiments indicates that the flow processes in the coupled soil-plant systems were well represented in the model.

By knowing the distribution of soil and root water potentials, the combined method presented here would allow to study the direct relation between water use and root or plant growth, as was recently shown by Bao et al. (2014). Nevertheless, this is the first study in which 3-D simulations of water flow in coupled soil-plant studies were performed based on real data of the root architecture and validated against measurements of soil water potential. We did not focus on how to setup an experiment so that root properties and their uncertainty could be derived from such a setup but we rather consider the study as a proof-of-concept. In future studies, inverse modeling could be carried out to determine the root parameters and their uncertainty.

## Author contributions

NK acquired and analyzed the experimental data, KH did the computational modeling. Both authors collaborated on the writing of the manuscript equally. EK did the setup of the computational modeling and the initial runs. JV, MJ, DV, and HV advised and commented on the manuscript.

### Conflict of interest statement

The authors declare that the research was conducted in the absence of any commercial or financial relationships that could be construed as a potential conflict of interest.

## Supplementary material

Supplementary Figure 1 **Development of soil matric potential ψ_ψ_ over time of the samples not used for modeling**. Different colors represent measurements in different depths/compartments.

Supplementary Figure 2 **Sums of the standard uptake fraction over soil depth increments of 0.25 cm for (A) the Split 1 root system at *t* = 30 days and (B) the Split 3 root system at *t* = 34 days solved for different parameterizations of radial and axial root hydraulic conductivities**.

Supplementary Figure 3 **Influence of paraffin layer on root growth: roots grow either unimpeded (left), but can also be deflected within the soft paraffin and later re-penetrate the soil**. Split 1, Day 12, Layer at -5 cm, Height of image section: 13.5 mm.

Supplementary Figure 4 ***Split 1* scenario *SC*: **(A)** line shows the mean and shaded areas the range (min - max) of soil water potential within each of the four soil compartments, **(B)** single slice at *z* = −12 cm showing gradients of soil water potential around the roots (black circles)**.
