# Supplementary figures and images for: Unraveling the hydrodynamics of split root water uptake experiments using CT scanned root architectures and three dimensional flow simulations
Source: Front Plant Sci. 2015 May 29;6:370. doi: 10.3389/fpls.2015.00370 (PMC4448007; doi:10.3389/fpls.2015.00370)

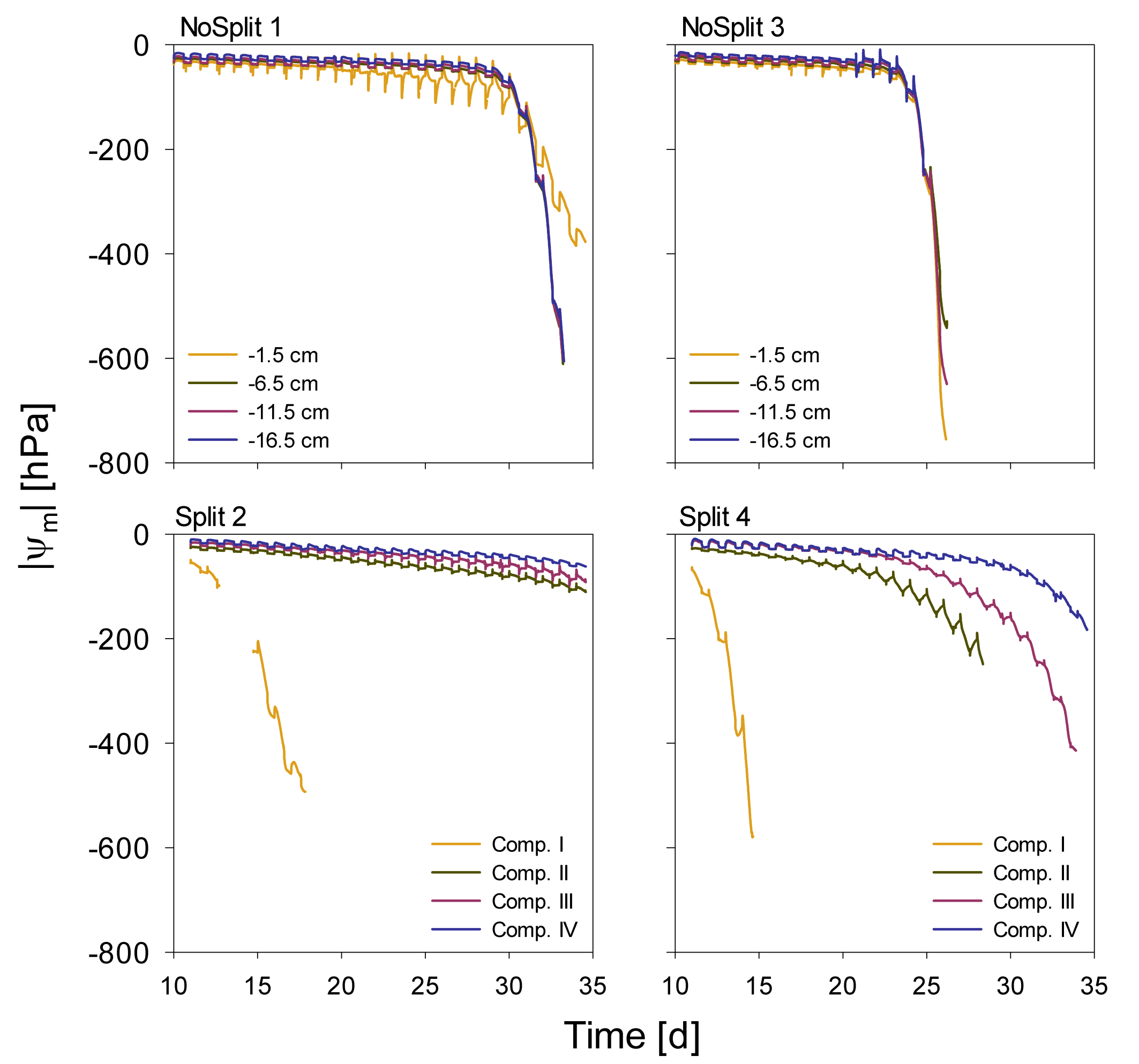

Supplement: Supplementary Figure 1 — Development of soil matric potential ψψ over time of the samples not used for modeling. Different colors represent measurements in different depths/compartments. [file Image1.TIF]

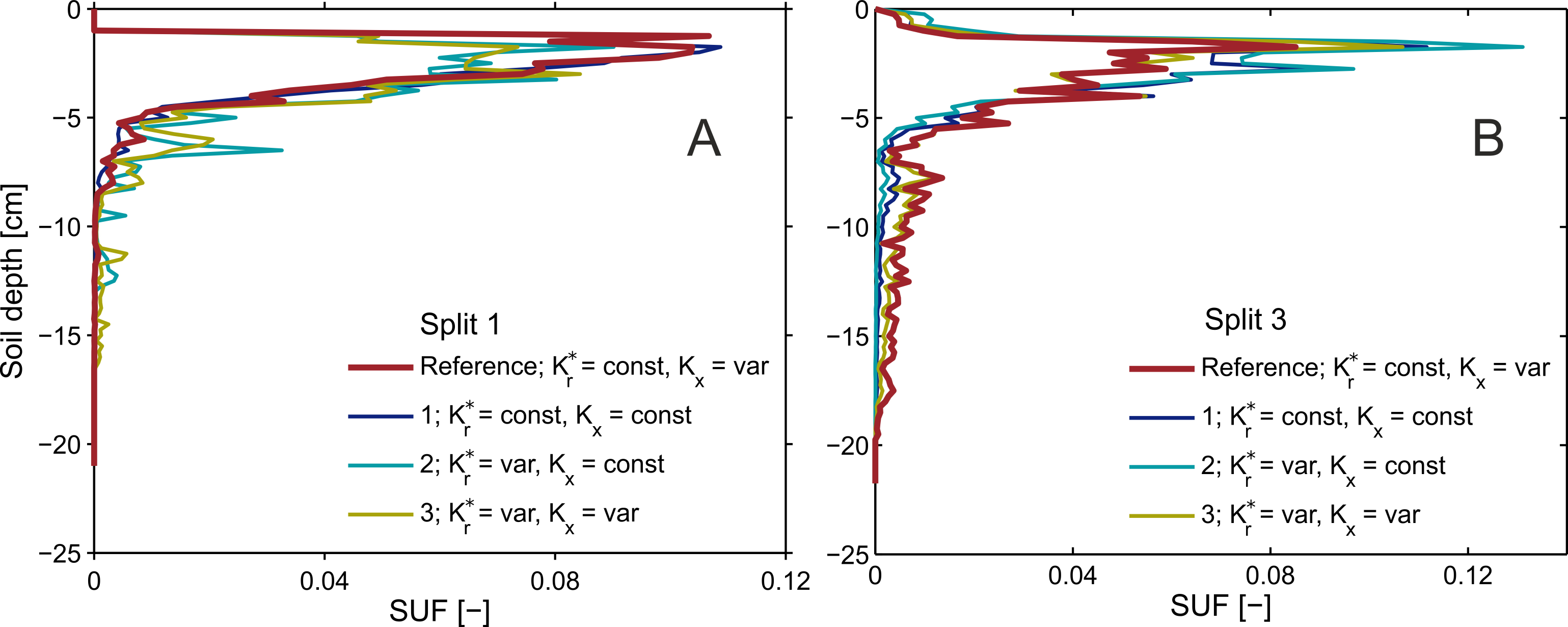

Supplement: Supplementary Figure 2 — Sums of the standard uptake fraction over soil depth increments of 0.25 cm for (A) the Split 1 root system at t = 30 days and (B) the Split 3 root system at t = 34 days solved for different parameterizations of radial and axial root hydraulic conductivities. [file Image2.TIF]

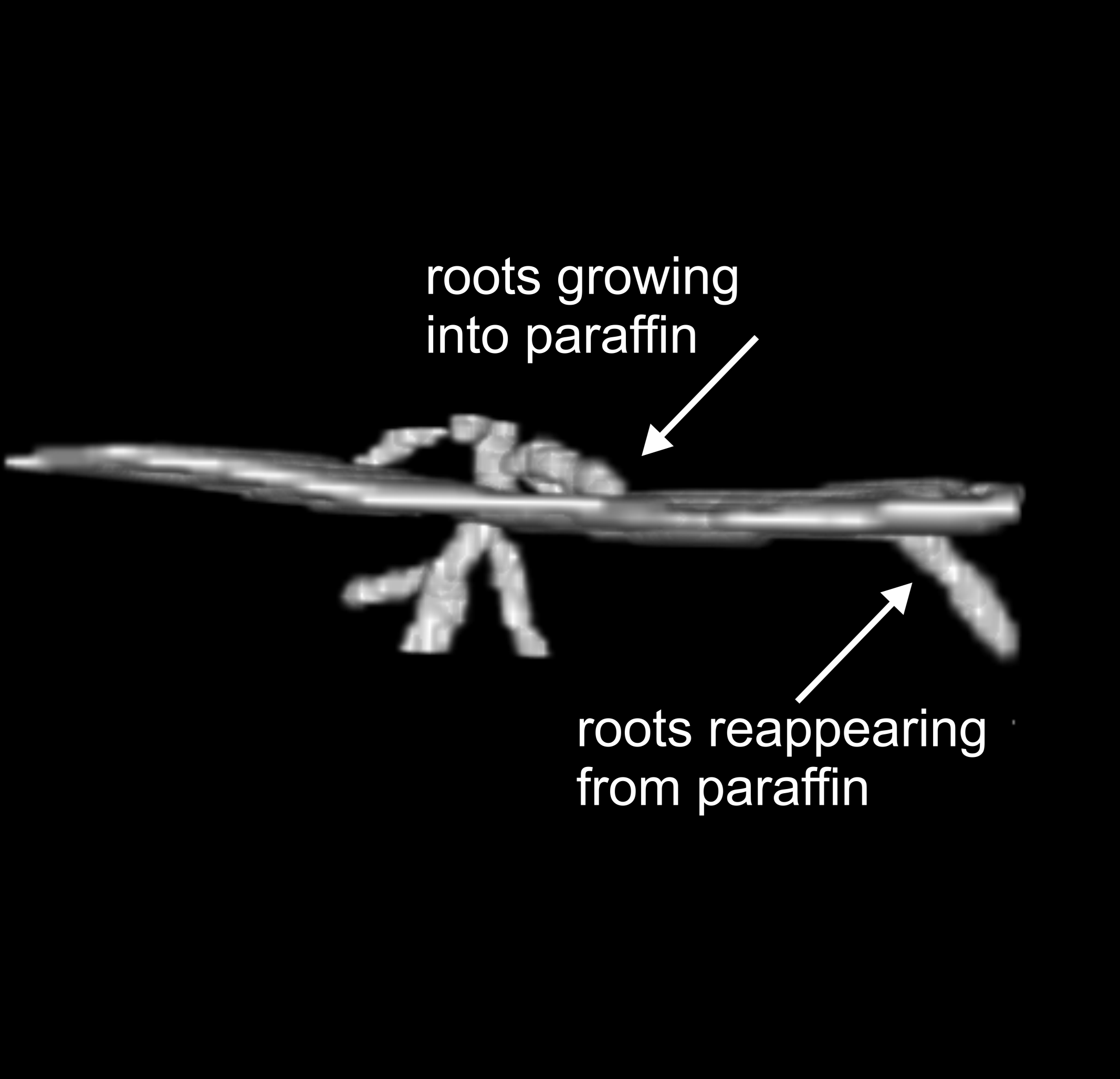

Supplement: Supplementary Figure 3 — Influence of paraffin layer on root growth: roots grow either unimpeded (left), but can also be deflected within the soft paraffin and later re-penetrate the soil. Split 1, Day 12, Layer at -5 cm, Height of image section: 13.5 mm. [file Image3.TIF]

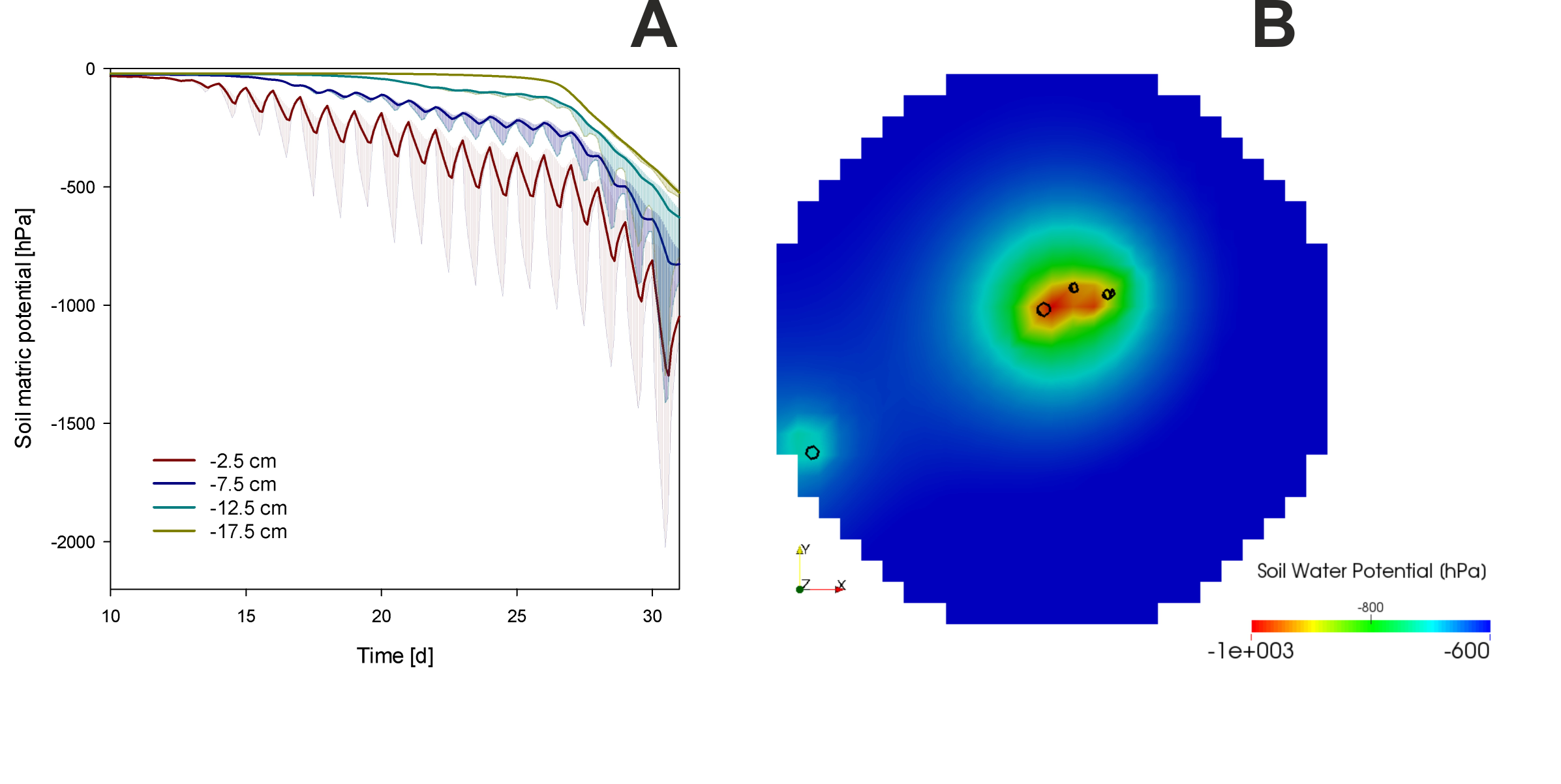

Supplement: Supplementary Figure 4 — Split 1 scenario SC: (A) line shows the mean and shaded areas the range (min - max) of soil water potential within each of the four soil compartments, (B) single slice at z = −12 cm showing gradients of soil water potential around the roots (black circles). [file Image4.TIF]
